# Supplementary material for: Patterns of Intron Gain and Loss in Fungi
Source: PLoS Biol. 2004 Nov 30;2(12):e422. doi: 10.1371/journal.pbio.0020422 (PMC532390; doi:10.1371/journal.pbio.0020422)
Supplement: Table S1 — Also available at http://genes.mit.edu/NielsenEtAl/. (4.3 MB ZIP). [file pbio.0020422.st001.zip › NielsenEtAl/html/803.html]

AN6817.1.NCU02299.1.MG10413.1.FG10920.1


```
 CLUSTAL W (1.82) Multiple Sequence Alignments - Introns Inserted


Sequence 1: MG10413.1	354 aa
Sequence 2: FG10920.1	349 aa
Sequence 3: NCU02299.1	379 aa
Sequence 4: AN6817.1	353 aa
Alignment Length: 390 aa
Number Identitical Residues: 114 aa
Alignment Score (without introns) 6707


MG10413.1 	-------MSRNNTSVVLAERPKPKIIPGKTF-AKKVQPAPTEADLKDGQILAETLYIGLD
NCU02299.1	MFHHTHGYKSHSLQLELVHRPSGPIIPGHTF-RVSAVPAPVPSDLNEGDLLLETLYISLD
FG10920.1 	-------MTRDNLEIVLAERPTTDIIPGRTF-TQKTGKAPTPADLQDGQILVETLYLSLD
AN6817.1  	---------MSNKQIILHERPTDAIDPSLTSGTFRLRTVPRPDNVPADKVLVRVHYLSLD
          	           . .: * .**.  * *. * .      .*   ::  ..:* .. *:.**

MG10413.1 	PAMRGWLND1ARSYVPPVQIDEVMRAAAVCRVLASRSKLAKR---GDLVSGMPGWQEISI
NCU02299.1	PAMRGWLDD~VRSYVPPVQIGEVMRAFCICRVLASRSPKAAA---GDIVTAMNGIREIGI
FG10920.1 	PAMRGWLRD1MRSYLPPVQIGETMRGSCACRVIASKSKLAKE---GDIVAATPGWTEFAI
AN6817.1  	PAMRQWLSA~SRSYIAPVKIGAVMRGVSMSRVVAVGKDLASKFQVGDWVSAYTGWQEYAV
          	**** **    ***:.**:*. .**. . .**:*  .  *   . ** *:.  *  * .:

MG10413.1 	LSEKGPRSFDAPFAVPPNGRPTDMLGVLGMTGLTAYMGVKHVAAIKPGDTVVVSGAAGAT
NCU02299.1	LSD---RHIEKPAFLPPGGKTTDLLGVLGMTGLTAYFGMMKIGNVKRGDTVVVSAAAGAT
FG10920.1 	VPE---GRFEPASYYPGVQDPKDMLSALGMTSLTAWVGMAKIADPQPGELVVVSGAAGAT
AN6817.1  	LGK---DDVEK-IAIPVGGRPTDVLSVLGLTGLTAYFGMIEIGQPRPGDTVVVSGAAGAT
          	: .     .:     *    ..*:*..**:*.***:.*: .:.  : *: ****.*****

MG10413.1 	GSVVGQMAKHVYGAGRVVGIAGGEDKCRWLVEELGFDAAIDYKAPDFKKKFAEATKGFID
NCU02299.1	GSVAVQIARIQG-AKRVVGTAGSDEKCRWLVEEGGADVALNYKDPEFRRKFKEATPDFVD
FG10920.1 	GSVAGQIAKLKG--ATVVGIAGGEDKCKWLTEELGFDVALNYKADDFKEKFNEATKNYID
AN6817.1  	GMVAGQIAKIKG-AKRVIGLAGTQDKCDFLVKELGFDVAINYKSPNWRKQLKAATPDYIN
          	* *. *:*:    :  *:* ** ::** :*.:* * *.*::**  :::.::  ** .:::

MG10413.1 	VYWDNV1GGEILDLALARAKKHARFVMCGGISTYNDSANEVRGP----------------
NCU02299.1	VYFDNV1GGEQLNMALARANRGARFVMCGGISQYN--AEHKQGPKVGGFHLLGTISAMEC
FG10920.1 	VYYDNV1GGDILDMCLMRAKEHARFIECGLVSQYN--AAKPQGP----------------
AN6817.1  	VYFDNV~GGEILDACLARAAPFSRFVMCGAISQYN--TSKPKGP----------------
          	**:*** **: *: .* **   :**: ** :* **  : . :**                

MG10413.1 	-----K0NINMVI~AMSIRMQGFIVFDYIKEYAQARQDLAKWLQEGKIKRGETIVKGGMD
NCU02299.1	STDKFQ~NINNVI~TMRIRMEGFIVFDYKDEYPTALGQLAQWLAEGKLKRKETILKGGVR
FG10920.1 	-----R~AFSQIV~TMRIKMQGFIVLDHAKDFATARADLAKWMSEGKLKKTETVIRGGLK
AN6817.1  	-----A~SYMNVI0SQRVTMKGFIVFDFAEKYAAAREELSSWLAQGKLARKDHILTGGLE
          	           :: :  : *:****:*. ..:. *  :*:.*: :**: : : :: **: 

MG10413.1 	VIEQTLVDLFNGVNKG1KLLVEVK----NPDSAKL
NCU02299.1	VADEGMVQLYKGGNIG1KLLIEVKNP-DEERQSRL
FG10920.1 	VAEQALVDLYKGINTG~KLIVEIKN--HNDTPSKL
AN6817.1  	SAPKALVDLYAGANTG~KMMVEVAPVNETEPRAKL
          	   : :*:*: * * * *:::*:   .     ::*
```
